# Measuring the Subjective Passage of Time: A Sociophysics Modeling

**DOI:** 10.3390/e26060528

**Published:** 2024-06-19

**Authors:** Serge Galam

**Affiliations:** CEVIPOF—Centre for Political Research, Sciences Po and CNRS, 1, Place Saint Thomas d’Aquin, 75007 Paris, France; serge.galam@sciencespo.fr

**Keywords:** subjective time, passage of time, past and future horizons, modeling, sociophysics

## Abstract

A simple model is built to evaluate quantitatively the individual feeling of the passage of time using a sociophysics approach. Given an objective unit of time like the year, I introduce an individualized mirror-subjective counterpart, which is inversely proportional to the number of objective units of time already experienced by a person. An associated duration of time is then calculated. Past and future individual horizons are also defined together with a subjective speed of time. Furthermore, I rescale the subjective unit of time by activating additional clocks connected to ritualized socializations, which mark and shape the specific times of an individual throughout their life. The model shows that without any ritual socialization, an individual perceives their anticipated life as infinite via a “soft” infinity. The past horizon is also perceived at infinity but with a “hard” infinity. However, the price for the first ritualized socialization is to exit eternity in terms of the anticipated future with the simultaneous reward of experiencing a finite moment of infinity analogous to that related to birth. I then extend the model using a power law of the number of past objective units of time to mitigate the phenomenon of shrinking of time. The findings are sound and recover common feelings about the passage of time over a lifetime. In particular, the fact that time passes more quickly with aging with a concomitant slowing down of the speed of time.

## 1. Introduction

Clocks are everywhere, shaping everyone’s path of life at both individual and collective levels. The goal was to construct a universal time frame that is independent of individuals and their histories in order to allow coordination among people who are located at different places at different times. Within this time frame, all events become connected to each other in a linear and orderly manner. Time is then the same for everyone, whether an individual or a machine. And indeed, that brings order to the “disorder” of times.

The associated “objective” time is measured mainly using seconds, minutes, hours, weeks, months, years, centuries, and millennia. Those units of time are fixed and unaffected by the passage of time itself. Moreover, the measure of the objective time has reached an incredible precision with the cesium atomic clocks, which serve as the world standard [1].

A major interest of these units lies in their ability to be counted and added objectively, systematically, everywhere, and, above all, understood and synchronized by everyone. Such an objective is achievable using one common counting origin. While the choice of an origin is arbitrary, it requires to be acknowledged and shared both individually and collectively to establish a unique metric of time.

Today, the Western calendar is the world-time secular norm being used worldwide and denoted the common era (CE) [2]. However, other origins of time and calendars exist and could have been chosen as well [3].

Nevertheless, while society as a whole, as well as individuals, are deploying within this universal standardization of time, an essential feature of the passage of time has yet to be elucidated.

It happens that the individual feeling of the perception of the passage of time differs from the objective passage of time measured by clocks. Accordingly, a unit of time like a day, a week, a month, or a year is perceived differently by individuals all along their lives. I thus associate a subjective mirror unit of time attached to each objective unit of time. A specific subjective mirror unit of time is attached to each person, and it varies with the time already experienced by this person [4]. In particular, the feeling of a shrinking of time with aging has been reported and established qualitatively long ago [5,6,7,8,9].

In the seventies, two attempts were made to mathematically quantify this duality [10,11]. Both used continuous time, postulating that the derivative of subjective time is proportional to the derivative of objective time divided by respectively the total objective time and the total subjective time experienced by a person. Those choices yield a logarithmic [10] and a square root of the total objective elapsed time for the total past subjective time [11]. Latter, a mathematical toy model recovers a logarithmic dependance using the dynamics of boxes being filled by balls [12]. The origin of the discrepancy phenomenon in time perception has also been linked to brain aging and associated cognitive abilities [13,14,15,16].

In this paper, going back to those earlier modeling attempts [10,11], I take up the whole problem again from the start [17]. Given a discrete objective unit of time like the year, my starting hypothesis introduces for each person a mirror subjective unit of time, which is inversely proportional to the number of objective units already experienced by that person. The counting of this number starts from their respective births till the present time. Therefore, the subjective unit of time gets smaller after each new lived objective unit.

A mirror subjective duration of the objective duration of a time interval is then obtained. I also define three related entities with a past horizon, a future horizon, and a speed of time. Furthermore, I extend the inversely proportional dependence to a power law dependence, allowing me to mitigate the amplitude of the shrinking effect along aging. The associated exponent could be an adjustable parameter to fit experimental studies.

Moreover, similarly to the birth event, I assign extra clocks to some socially ritualized events. Then, stacking those individual additional clocks rescales the subjective unit of time specific to each person.

The results shed a novel light on the phenomenon of subjective passage of time by identifying a series of universal features, which allow to quantitative estimates at individual levels. Common associated feelings are also recovered. In particular, the fact that time seems to pass more quickly with age.

By building a model of the subjective passage of time at an individual level from simple hypotheses using a sociophysics approach [18,19,20,21,22,23], where, to my knowledge, the topic has not been investigated so far, contrary to philosophy [24,25], psychology [6,26,27,28,29], and neuroscience [14].

The rest of the paper is organized as follows: The definition of a subjective unit of time associated with an objective unit of time is presented in Section 2. Section 3 defines the subjective perception of a duration of an interval of objective time. A speed for the subjective passage of time is also defined. Past and future horizons are introduced in Section 4. A power law for the subjective unit of time is suggested in Section 5. The creation of an additional clock associated with the setting of a ritualized socialization is analyzed in Section 6. The process is repeated with a stacking of clocks in Section 7. Section 8 sums up the results with concluding remarks.

## 2. At the Beginning Stands an Observation

The time clock is omnipresent in everyone’s life and understood by all. It regulates all moments of life at the individual and collective levels. The measured time is deployed mainly along seconds, minutes, hours, days, weeks, months, years, decades, centuries, and millennia. I denote $U_{o}$ any one of these various objective units. In the following, I select the year for this objective unit to help illustrate the presentation.

While a year is perfectly well defined for everyone, its duration is perceived differently at the individual subjective level from one person to another and at different times for the same person. To introduce an element that is common to all but specific to each individual, I consider an individual’s lived experience in terms of their total number of years lived.

Thus, for a one-year-old child, a year will be their whole life, while for a six-month-old infant, a year is completely undefined, and for a hundred-year-old adult, it is only one hundredth of their life. Thus, every individual can define objectively a subjective unit of time by apprehending their full life as a unitary whole in terms of the chosen objective unit of time. This observation is a basic fact accessible to all, like Newton’s falling apple. It was evoked almost 150 years ago to justify the existence of a subjective passage of time in opposition to the objective passage of time [5].

**Figure 1:**
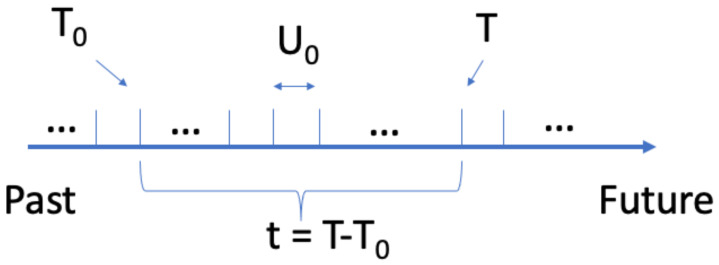
Schematic representation of the linear objective time. $T_{0}$ is the birthdate of a given person, *T* is the current time, and $U_{o}$ is an objective unit of time.

To capture the above feature into a mathematical formula applicable to each person, I first denote $T_{0}$ the date of birth of a person along the CE calendar. The variable *t* counts the number of passed objective units of time $U_{o}$ at date *T*. Thus, at date *T*, a person born at $T_{0}$ has lived $t=T-T_{0}$ units $U_{o}$ , here years as shown in Figure 1.

**Figure 2:**
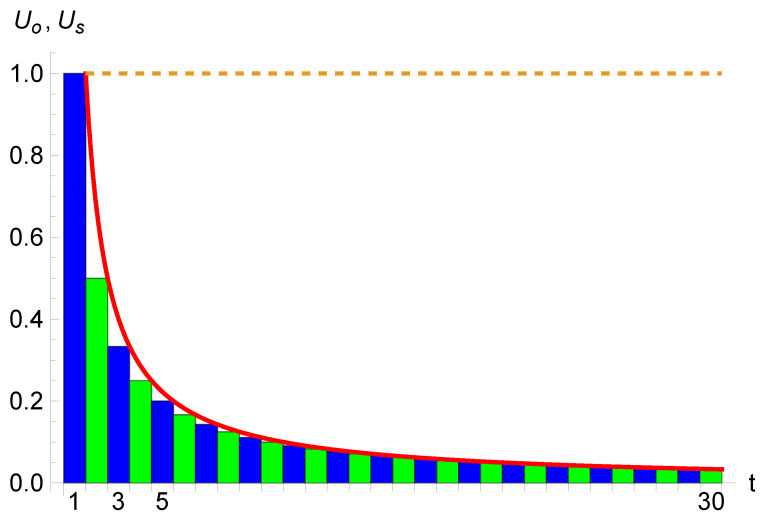
The variation of the subjective unit of time $U_{s}$ as a function of the number $t=T-T_{0}$ of passed objective units of time $U_{o}$ . For t=1 $U_{s}=U_{o}$ , but for t=20 , $U_{s}=0.05U_{o}$ and $U_{s}=0.03U_{o}$ for t=30 . The infinite value of $U_{s}$ during the period preceding the passage of one unit of objective time is not shown. The curve exhibits the variation for continuous time *t*. The vertical dotted line shows the objective unit of time $U_{o}$ , which is by definition constant over time.

Therefore, the subjective perception of the chosen objective unit $U_{o}$ is defined as

(1) $$U_{s}=\frac{1}{t}U_{o},$$

which makes the unit $U_{s}$ to vary from one individual to another via the counting origin $T_{0}$ and the current moment of evaluation via *t*. From Equation (1), we recover the above starting observation that for t=1 , $U_{s}=U_{o}$ and for t=10 , $U_{s}=0.1U_{o}$ . Figure 2 shows the variation of $U_{s}$ as a function of the number of passed units $U_{o}$ . The shrinking of the subjective unit of time is rather rapid and significant.

Moreover, it is interesting to note that for the period $T_{0}<T<T_{0}+1\Rightarrow T=T_{0}$ , which gives t=0 and thus an infinite unit $U_{s}\to +\infty$ . As a consequence, during the passage of time preceding the completion of the first objective unit of time, the mirror subjective unit is perceived as infinite. During a child’s first year of life, their related perception of time is infinity.

It is the discretization of time [30], in terms of objective units, that produces this effect of an initial infinite time perception. A continuous time erases the singularity of Equation (1) at t=0 since then t≠0 at once. While discrete time creates a staircase curve, continuous time makes the curve smooth, as seen in Figure 2.

## 3. Duration of Time: Subjective versus Objective Perceptions

Given two dates $T_{1}$ and $T_{2}>T_{1}$ , the duration of time elapsed between $T_{1}$ and $T_{2}$ writes

(2) $$D_{12}=T_{2}-T_{1},$$

which corresponds to a number of objective units,

(3) $$d_{12}=\left(t_{2}-t_{1}\right)U_{o},$$

where $t_{1,2}=\left(T_{1,2}-T_{0}\right)$ .

### 3.1. Subjective Duration

To calculate the subjective mirror of $d_{12}$ using Equation (1), I evaluate both subjective durations to reach respectively $T_{1}$ and $T_{2}$ with,

(4) $$ds_{1,2}=\sum_{t=1}^{t_{1,2}}\frac{1}{t}U_{o},$$

which can be written as

(5) $$ds_{1,2}=H_{t_{1,2}}U_{o},$$

where $H_{t_{1,2}}$ are $t_{1,2}$ -th harmonic numbers. The subjective mirror duration is thus,

(6) $$ds_{12}=ds_{2}-ds_{1}=\sum_{t=t_{1}+1}^{t_{2}}\frac{1}{t}U_{o}.$$

which can be rewritten $ds_{12}=\left(H_{t_{2}}-H_{t_{1}}\right)U_{o}$ .

At this point, it is worth mentioning that a purely mathematical toy model with boxes and balls [12] recovered a logarithmic dependence for the duration of time. The evaluation is done by calculating the numbers of balls required to fill M boxed with at least one ball given a number N of boxes. The balls are associated with events in individual lives, and the number of boxes corresponds to the lengths of those lives. Time duration is then obtained as the ratio M/N. Indeed, putting $N=t_{2}$ and $t_{1}=N-M+1$ yields exactly Equation (6) without the considered current objective unit of time. It shows that to cover a period of time $t_{2}-t_{1}$ requires to fill M boxes with at least one ball. The mathematical formula does not specify the unit of time being used.

It is also worth noting that making the time continuous turns the duration $ds_{12}$ logarithmic, as seen from

(7) $$ds_{12c}=\int_{t_{1}}^{t_{2}}\frac{1}{t}U_{o}=\ln\left(\frac{t_{2}}{t_{1}}\right)U_{o},$$

thus recovering a previous result derived from Equation (1) with *t* being a continuous variable [10].

**Figure 3:**
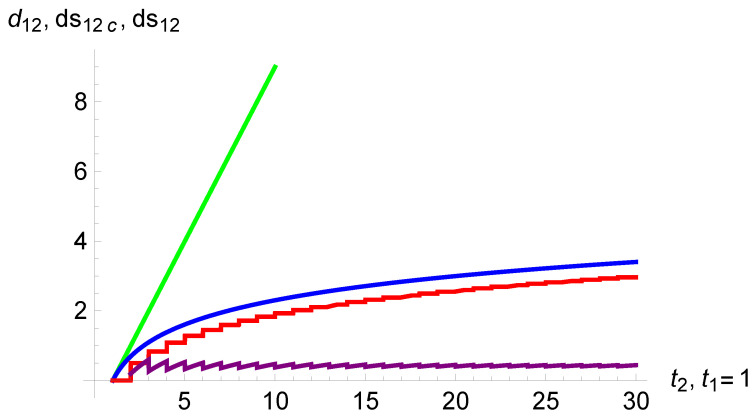
The variation of the subjective duration of time as a function of the objective duration $t_{2}$ from $t_{1}=1$ . Equations (6) and (7) are shown by the staircase (red) and smooth (blue) curves respectively. The lower staircase (purple) curve exhibits the difference between the two curves. The straight line (green) represents the objective duration of time.

Figure 3 illustrates the shrinking of the subjective duration of time as a function of the related objective duration for both Equations (6) and (7) as a function of $t_{2}$ from $t_{1}=1$ . The difference between them is also shown and found to be very small, with a constant value around 0.42 .

### 3.2. Multiplicative Shrinking

The logarithmic form of Equation (7) hints at a pattern in the shrinking of subjective duration with the passage of time. Indeed, multiplying simultaneously $t_{1}$ and $t_{2}$ by any integer *k* leaves $ds_{12}$ invariant, which is a remarkable property of the dynamic of shrinking of time perception.

For instance, given two intervals of time duration $t_{2}-t_{1}$ and $t_{4}-t_{3}$ with $t_{2}=kt_{1}$ and $t_{4}=kt_{3}$ , it gives the same duration with $ds_{12}=ds_{34}=\ln\left(k\right)U_{o}$ , whatever the values of $t_{1}$ and $t_{3}$ . However, the associate objective durations $t_{2}-t_{1}=\left(k-1\right)t_{1}$ and $t_{4}-t_{3}=\left(k-1\right)t_{3}$ are not equal.

Therefore, multiplying an interval of objective time $t_{2}-t_{1}=\left(k-1\right)t_{1}$ by any integer *l* keeps the subjective duration unchanged with $\ln\left(k\right)U_{o}$ . In contrast, the associated objective duration is multiplied by *l* with the interval $l(t_{2}-t_{1})$ .

For example, intervals from 6 to 12, 12 to 24, 24 to 48, and 48 to 96, i.e., durations of 6, 12, 24, 48 years, are perceived subjectively as equal in duration with ln(2)≈0.69 year, thus exhibiting a significant shrinkage with aging. Equivalently, intervals from 5 to 15 and from 20 to 60, i.e., 10 and 40 years, are perceived equally with ln(3)≈1.10 year.

### 3.3. Paradoxical Feelings: Duration versus Speed

The above results support the common feeling that the perception of the passage of time shrinks with aging. However, that relates to the perception of an elapsed time interval and not to the speed at which time passes. In order to address the question of the speed at which a person perceives the passage of time, I now define the subjective speed of time $vs_{1,2}$ as follows:

(8) $$ds_{12}=vs_{12}d_{12},$$

where $ds_{1,2}$ is the subjective duration of the objective $d_{1,2}$ time interval. Using Equations (6) and (7) leads to

(9) $$vs_{12}=\frac{1}{t_{2}-t_{1}}\sum_{t=t_{1}+1}^{t_{2}}\frac{1}{t},$$

for discrete time and

(10) $$vs_{12c}=\frac{1}{t_{2}-t_{1}}\ln\left(\frac{t_{2}}{t_{1}}\right),$$

for continuous time.

**Figure 4:**
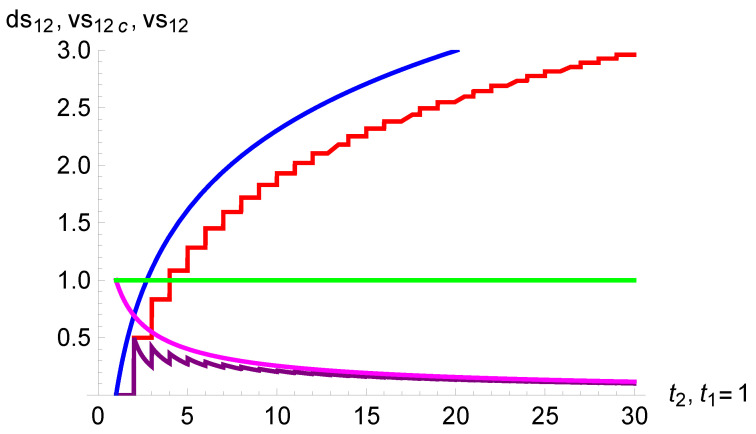
The variation of the subjective speed of time as a function of the objective duration $t_{2}$ from $t_{1}=1$ . Equations (9) and (10) are shown by the staircase (purple) and smooth (magenta) curves, respectively. The upper curve exhibits the durations $ds_{12}$ (staircase, red) and $ds_{12c}$ (smooth, blue). The horizontal line (green) is the objective speed, which is constant and equal to one.

Figure 4 shows the variation of both discrete and continuous subjective speeds of time $vs_{12}$ and $vs_{12c}$ as a function of the objective duration $t_{2}$ from $t_{1}=1$ . The durations $ds_{12}$ and $ds_{12c}$ are also shown. As above, the difference between discrete and continuous times is rather small from $t_{2}>10$ , as seen in the figure. The objective speed is constant and equal to one.

The results show that the shorter is the perception of the duration of time, the slower is the perception of the speed at which the time passes. The shrinking of time duration is thus concomitant to a slowing down of the speed of time. That provides an explanation to the paradoxical feeling about the subjective feeling of the passage of time for young versus old people. For the first group, time flies and duration is long, while for the second group, time gets almost frozen but yet duration is short. Intuitively, this result can be understood with the analogy of a traveling car. Driving at a subjective speed during an objective duration yields the travelled subjective duration. Therefore, the slower is the speed, and the shorter is the elapsed subjective duration to travel over an objective duration of time.

## 4. Past and Future Horizons

At this stage, two additional functions can be defined in connection to the global subjective perception of the passage of time for each person. I denote the first one past horizon and the second one by future horizon.

### 4.1. Past Horizon

The past horizon $H_{p}$ is obtained when an individual turns to the past, looking at their horizon of the past at present time *T*. In doing so, they add up backwards all their subjective years already lived since their birth at $T_{0}$ . The past horizon, thus writes

(11) $$H_{p}=\sum_{k=0}^{t}\frac{1}{t-k}U_{o}.$$

For instance, an individual born in 2004 at time T=2024 has a past horizon,

(12) $$\begin{array}{ccc} H_{p} & = & \left\{\frac{1}{20}+\frac{1}{19}+\frac{1}{18}+\cdots +\frac{1}{1}+\frac{1}{0}\right\}U_{o},\\ & = & \left\{0.05+0.053+0.056\cdots +1+\infty \right\}U_{o},\\ & \approx & \left\{3.598+\infty \right\}U_{o}, \end{array}$$

where the number of terms in the sum is finite but the total is infinite with $H_{p}\to +\infty$ . However, it is only the last term of the sum $\frac{1}{0}$ at t=0 (birth) that corresponds to the first experimentation of the unit of time, which creates the limit to infinity. To account for the psychological dimension of the perception of this infinity as being sudden in a single jump from one, I denote it a “hard” infinity.

**Figure 5:**
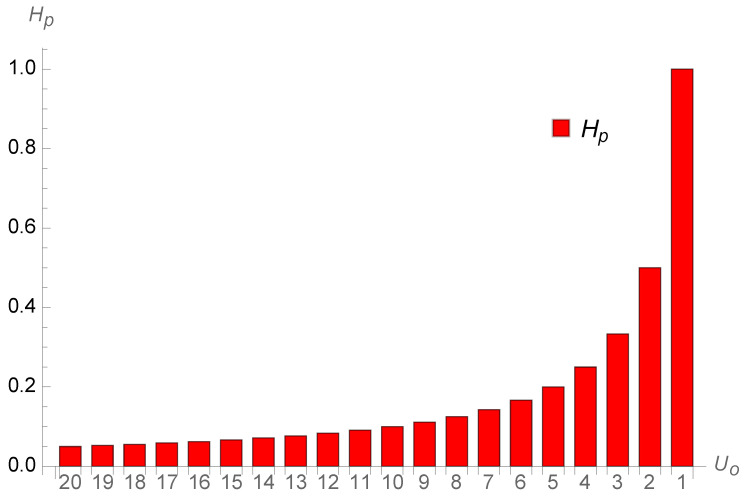
All single contributions from each past year without the infinite first one. Each term gets larger till one just before infinity.

Figure 5 shows all single contributions from each past year without the infinite first one. Each term gets larger till one just before infinity when looking back at the past.

### 4.2. Future Horizon

The future horizon $H_{f1}$ is the duration of time perceived by a person who projects themselves into the future to reach a specific deadline. To calculate the subjective evaluation of this time, I assume that at present time *T*, when an individual born at $T_{0}$ looks ahead at the time horizon encompassing the next *m* years, they automatically add up all the related subjective coming *m* years from this moment. At time *T*, the future horizon for the next *m* years thus writes

(13) $$H_{f}=\sum_{k=1}^{m}\frac{1}{t+k}U_{o},$$

which can be rewritten as

(14) $$H_{f}=\left[\sum_{k=1}^{t+m}\frac{1}{k}-\sum_{k=1}^{t}\frac{1}{k}\right]U_{o},$$

yielding the difference between two harmonic numbers, $H_{t+m}$ and $H_{t}$ . Shifting to continuous time, I get,

(15) $$H_{fc}=\log\left(\frac{t+m}{t}\right)U_{o}.$$

The variation of $H_{f}$ is found to be paradoxical as a function of *m*. For instance, a person born at $T_{0}=2004$ looking at time T=2024 at 10 years ahead, their objective age is 20 years, and their related perceived future horizon equals

(16) $$\begin{array}{ccc} H_{f} & = & \left\{\frac{1}{20}+\frac{1}{21}+\frac{1}{22}+\cdots +\frac{1}{30}\right\}U_{o},\\ & = & \left\{0.05+0.048+0.045\cdots +0.033\right\}U_{o},\\ & \approx & 0.4472U_{o}, \end{array}$$

which is lower than one objective year.

**Figure 6:**
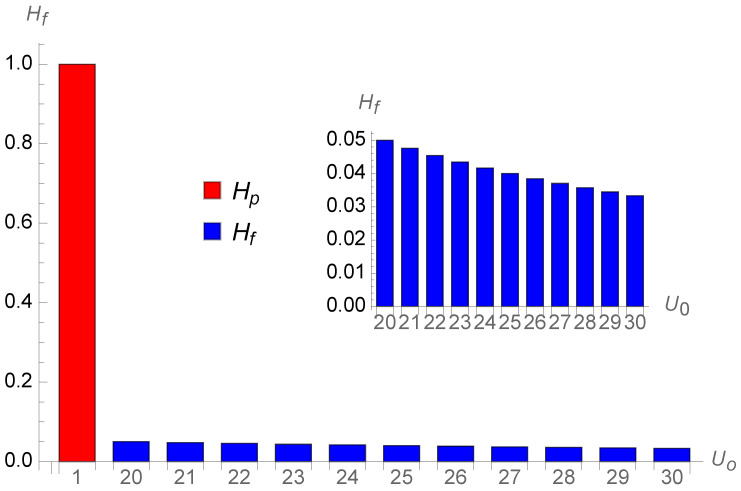
Successive single year contributions (in blue) for a twenty-year-old person looking ten years ahead. The first-year objective year (in red) is included to show the substantial shrinking of the subsequent subjective years; at twenty years old, the perception is only $0.05U_{o}$ . The inset exhibits the same content without the objective unit.

Figure 6 shows the successive single contributions from each additional future year. The first-year objective year is included to show the substantial drop of the subsequent subjective years; at twenty years old, the perception is only $0.05U_{o}$ . The inset exhibits the same content without the objective unit.

However, for the same person looking at the future without a specific date, Equation (16) becomes,

(17) $$H_{f}=\left[\frac{1}{20}+\frac{1}{21}+\frac{1}{22}+\cdots +\frac{1}{30}+\cdots +\frac{1}{\infty }\right]U_{o},$$

which includes an infinite number of years since they have no a priori reason to set an upper limit on their life in terms of longevity. People do not include a date for their own death.

Accordingly, although each new term $\frac{1}{t+k}$ added becomes smaller and smaller since (t+k) becomes larger and larger, the number of terms added grows faster than $\frac{1}{t+k}$ decreases. As a result, the associated future horizon tends to infinity ( $H_{f}\to +\infty$ ), despite having every additional subjective year tending to zero.

**Figure 7:**
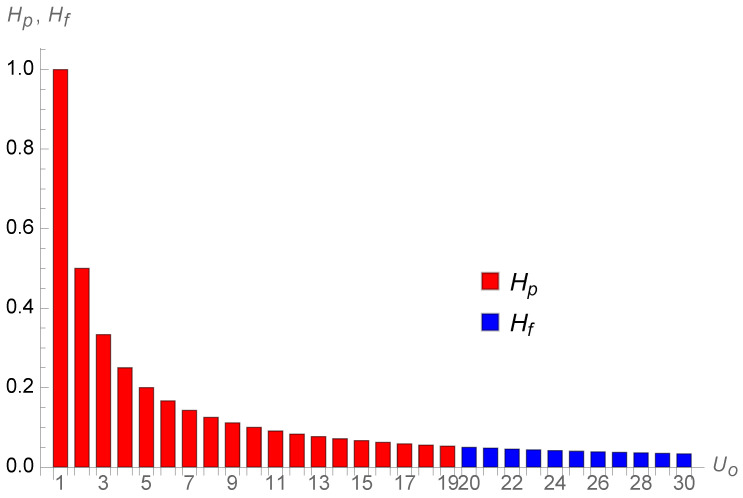
Both past horizon (in red) without the infinite initial term and future horizon (in blue) for a finite time projection at ten years ahead.

Consequently, the psychological perception of this infinity is smooth and unlocalized. It is fuzzy. To account for this blurred characteristic of the perception of infinity that contrasts with the “hard” infinity defined above, I call it the “soft” infinity. Figure 7 includes both the past horizon (without the infinite initial term) and the future horizon for a finite time projection at ten years ahead.

## 5. A Power Law to Mitigate the Effect of Proportional Reduction

The above results and trends are sound with respect to observations related to the various aspects of the subjective perception of the passage of time. However, they may be quantitatively inappropriate. In particular, the shrinking magnitude of $U_{s}$ seems too drastic in amplitude with the increasing number *t* of years lived. Indeed, it is sound to consider that after a certain number of years already past for a person, the associated proportionality feeling fades with a much weaker effect on the value of the mirror unit. Therefore, damping the dynamics of shrinking may be in order to make the framework more realistic.

One possibility to mitigate this effect is to extend the main hypothesis of proportionality of the current unit with respect to the number of elapsed objective units into a power law dependence. Accordingly, Equation (1) becomes

(18) $$U_{sa}=\frac{1}{t^{a}}U_{o},$$

with 0≤a≤1 .

**Figure 8:**
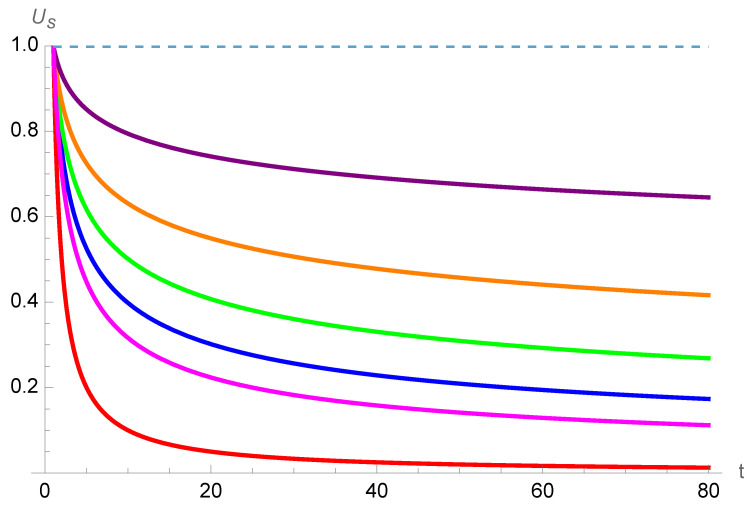
Variation of the subjective unit of time $U_{sa}$ as a function of the number of past objective *t* units of time $U_{o}$ for exponents a=1 (red), 0.5 (magenta), 0.4 (blue), 0.3 (green), 0.2 (orange), 0.1 (purple), 0 (dots).

Introducing an exponent allows to refrain the immediate shrinking of the perception of time, keeping the associated tail at a higher value. That reduction of the dumping opens a more flexible tackling of the phenomenon, as seen in Figure 8 for a series of values of the exponent. The power law reduces substantially the decreasing of $U_{s}$ with *t* as seen for a=1,0.5,0.4,0.3,0.2,0.1,0 . In addition, the value of the exponent *a* could become a fitting parameter in case a connection to some experiments would be feasible. Adding a degree of freedom allows either to assume the value of *a* is universal, being identical for everyone or specific to each person.

**Figure 9:**
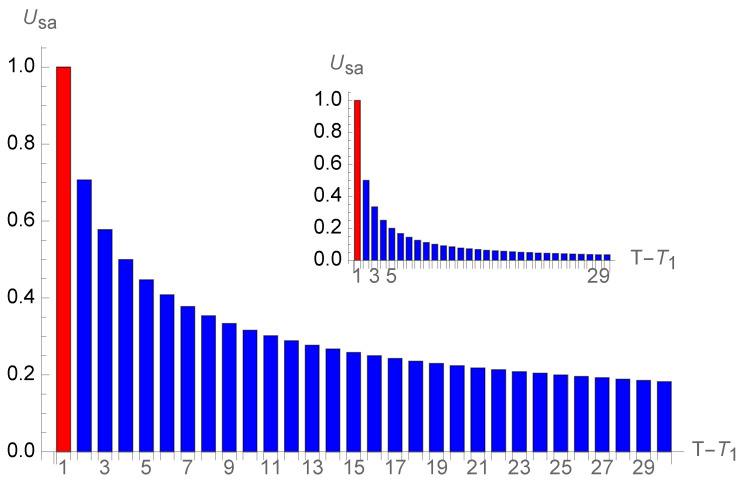
The variations of $U_{sa}$ for the first thirty years of existence of a person in the case a=0.5 , which means a square root dependence. The inset shows the variation of $U_{sa}$ for a=1 , which yields a stronger shrinking of the subjective unit of time.

To illustrate the damping effect, Figure 9 shows the variations of $U_{sa}$ for the first thirty years of existence of a person in the case a=0.5 , which means a square root dependence. The inset shows the variation of $U_{sa}$ for a=1 .

### Extended Subjective Duration

Using Equation (18) instead of Equation (1) modifies the subjective duration with

(19) $$dsa_{1,2}=\sum_{t=1}^{t_{1,2}}\frac{1}{t^{a}}U_{o},$$

which yields the extended subjective duration:

(20) $$dsa_{12}=dsa_{2}-dsa_{1}=\sum_{t=t_{1}+1}^{t_{2}}\frac{1}{t^{a}}U_{o}.$$

Shifting to continuous time turns the duration $dsa_{12}$ to

(21) $$dsa_{12c}=\int_{t_{1}}^{t_{2}}\frac{1}{t^{a}}U_{o}=\frac{t_{2}^{1-a}-t_{1}^{1-a}}{1-a}U_{o}.$$

It is worth noting that putting a=0.5 recovers the second previous attempt to model the subjective passage of time with $\frac{1}{\sqrt{t}}$ for the subjective unit of time after *t* objective units [11]. Indeed, Equation (18) extends both previous attempts at a=1 and a=0.5 , respectively [10,11].

The qualitative behavior of both past horizon and future horizon is preserved using the power law of Equation (18), but the quantitative values are modified with an attenuation of the shrinking effects.

## 6. Replicating Birth via Ritual Socialization

It is remarkable to note that all human societies have embedded a series of additional “births” via the practice of well-defined social rituals that ponder the social life of every person. These additional births are implemented via social rituals, such as communion for most religions, graduation during studies, military service in some countries, weddings, birth of children, and divorce. Indeed, what matters is the related initialization of an additional new counting of years, which starts with the setting of the associated event.

Ritualized social events are always small in number and occur mainly in the first thirty years of life. They shape the lifetime of each member of a collective community with a series of dates $T_{1},T_{2},T_{3},T_{4},\ldots$ , which punctuate the passage of time in everyone lifetime.

New counting clocks are thus “naturally” added to the $T_{0}$ initial birth clock, which marked the beginning of counting of objective lived time units. To incorporate these new clocks within the framework of the model, I consider the introduction of the first significant event at a date $T_{1}$ . Then, I assume that a second clock is created in addition and similarly to that following birth.

Replicating the same mechanism used to build the subjective unit $U_{s}$ from $U_{o}$ , I define a new subjective unit of time $U_{s1}$ from $U_{s}$ as

(22) $$U_{s1}=\frac{1}{t_{1}}U_{s},$$

where $t_{1}=T-T_{1}$ . Using Equation (1),

(23) $$U_{s1}=\frac{1}{tt_{1}}U_{o}.$$

**Figure 10:**
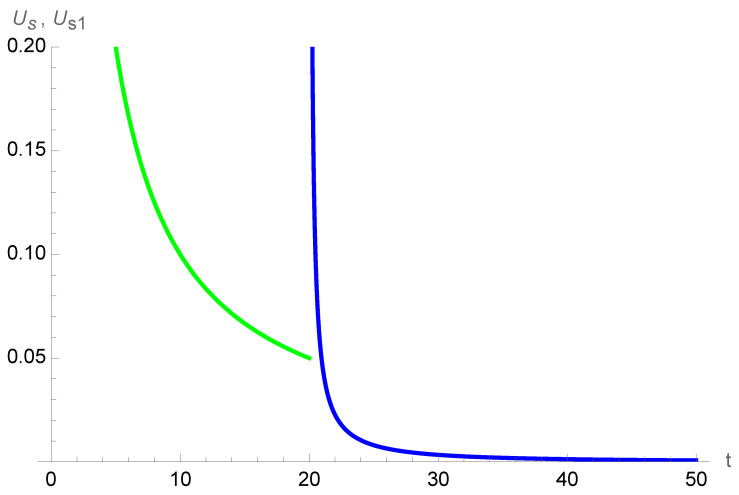
Variations of $U_{s}$ (in green) and $U_{s1}$ (in blue) for respectively the first twenty years (Equation (1)) and the following thirty years after a first social birth at age 20 (Equation (23)). While a singularity is seen at t=20 , the subsequent shrinking is amplified with respect to the previous one.

As before, during the period $T_{1}\leq T<T_{1}+1$ , $T=T_{1}\Rightarrow t_{1}=0$ , which reproduces the effect of hard infinity with $U_{s1}\to \infty$ . Thus, the reward from the first “socialization” through the ritual, for example, a wedding, is to be born again with the initial sensation of infinity felt during their first year of existence just after birth. Figure 10 shows the variation of Equation (23) during fifty years for a person with a social birth at age 20.

As a result, the individual is quite happy with the initiation of a second clock, on top of the first. The subsequent effect is the creation of a sequentially different subjective unit of time, which shrinks more quickly.

### 6.1. Past Horizon

With respect to the past horizon, at time *T* two different backward sums have now to be considered. The first one corresponds to the current unit $U_{s1}$ , from *T* back to $T_{1}$ , and the second one, with the subjective unit $U_{s}$ , from $T_{1}$ back to $T_{0}$ . Thus,

(24) $$H_{p1}=\left[\sum_{k=t-t_{1}}^{t}\frac{1}{k(k-t+t_{1})}+\sum_{k=0}^{t-t_{1}}\frac{1}{k}\right]U_{o}.$$

The sum is doubly infinite due to the two terms at $k=t_{1}$ and k=0 . The perception of an infinite origin is thus reactivated, but it becomes blurry with the feeling that the origin is even further away due to the addition of two different infinities at different times.

For the case of the individual born in 2004, if married at $T_{2}=2034$ , their past horizon at T=2044 would be,

(25) $$\begin{array}{ccc} H_{p1} & = & [\frac{1}{10\times 40}+\frac{1}{9\times 39}+\cdots +\frac{1}{1\times 31}+\frac{1}{0\times 30}\\ & + & \frac{1}{30}+\frac{1}{29}+\frac{1}{28}+\cdots \frac{1}{1}+\frac{1}{0}]U_{o},\\ & = & [0.0025+0.0028+\cdots +0.032+\infty \\ & + & 0.033+0.035+0.036+\cdots +1+\infty ]U_{o}\\ & = & \left[3.995+0.088+2\times +\infty \right]U_{o}. \end{array}$$

The outcome is thus similar to the case prior to the first ritual socialization, as seen when comparing with Equation (12).

### 6.2. Future Horizon

However, while the introduction of a second clock did not change qualitatively the perception of the past horizon, a qualitative change does occur for the future horizon $H_{f}$ , which writes

(26) $$H_{f1}=\sum_{k=1}^{m}\frac{1}{k(k-t+t_{1})}U_{o}.$$

for a projection in the coming *m* units of time from date *T*.

As before, when m→∞ , the sum is over an infinite series of decreasing terms. In contrast, now the successive terms vary as $\frac{1}{t^{2}}$ instead of $\frac{1}{t}$ , which in turn has a drastic impact on the limit. Indeed, it happens that $\frac{1}{t^{2}}$ tends faster to zero than the number of added terms, and thus the sum $H_{f1}$ no longer diverges towards infinity as $H_{f}$ does. The future horizon becomes bounded, i.e., its numerical value is finite.

The asymptotic difference appears clearly in the continuous version of the time. In this case, the horizon is obtained using the integrals of the subjective units $\frac{1}{t^{2}}$ and $\frac{1}{t}$ , which yield respectively $-\frac{1}{t}$ and logt , whose limits are 0 and +∞ for t→+∞ .

This qualitative change in the perception of time implies that the future horizon suddenly becomes visible. Projection in the “infinite” future is now at a finite distance from the present. The first ritualized socialization turns “eternal ”humans to “mortal” humans with respect to the perception of their ending future.

## 7. The Stacking of Clocks

The first clock-like socialization has created the condition to experience again the feeling of eternity during the completion of the following year. However, the associated cost was a reduction in the perception of the objective time remaining to live.

Empowered by this paradoxical life experience, social communities have been adding more social ritual to create additional clocks for the subjective passage of time. In doing so, individuals get the possibility of reiterating the experience of eternity during an objective unit of time, yet perceived as shorter and shorter at each new time, with the concomitant perception of an increasingly shorter time to live.

Accordingly, organized human groups have built a series of rituals that are quasi-mandatory to go through for each of their members. The net result is a stacking of several subjective clocks. The number *L* of ritual socializations and the associated clocks is a function of the various cultural communities and may also vary from one person to another within a given community.

Each new clock is set at a different time, yielding a series of social births at times $T_{2},T_{3}\ldots ,T_{L}$ , which results in a subjective unit of time written as

(27) $$U_{sL}=\frac{1}{\prod_{i=0}^{L}t_{i}}U_{o}.$$

where $t_{i}\equiv t-T_{i}$ and $T_{0}$ is the birthdate.

The subjective unit of time is now a function of $\frac{1}{t^{L}}$ , i.e., with a rapid convergence to zero. The larger *L*, the more abrupt this fall in the subjective duration. The choice of a stack of clocks is based on the fact that, at any given moment, people have one global sense of time passing, which de facto takes into account all their different clocks. At least, that is my hypothesis. Considering parallel feelings of time might have been an option, but at this stage, I found the stack simpler and more solid. These extra clocks are located in the memory of the related person. Some can also be located in formal contracts, such as a work contract, a wedding, or a divorce. They are activated throughout the cognitive perception of time.

### The Power Law Case

Using a power law for the mirror unit, Equation (27) becomes

(28) $$U_{saL}=\frac{1}{\prod_{i=0}^{L}t_{i}^{a}}U_{o},$$

which does mitigate the above effects as a function of the exponent *a*.

**Figure 11:**
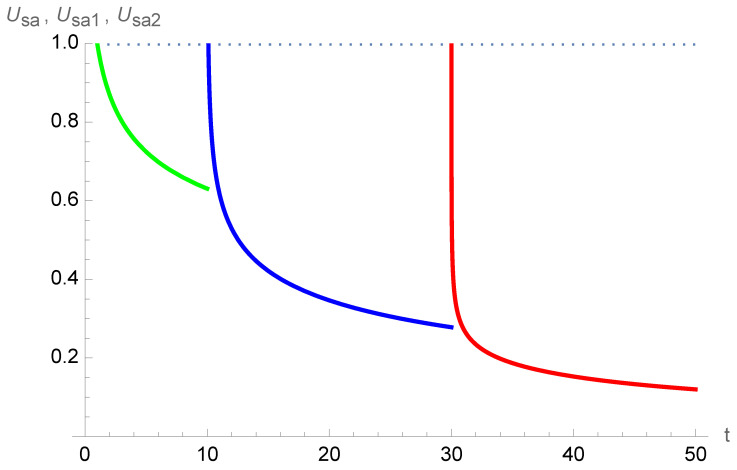
Case with two social births at ages 10 and 30, which produces two successive rescalings of the subjective unit of time $U_{sa}$ . The power law has an exponent a=0.20 . As a function of time, the subjective unit of time is $U_{sa}$ (green) for 0≤t≤10 , $U_{sa1}$ (blue) for 10≤t≤30 , and $U_{sa2}$ (red) for 30≤t≤50 . Although the shrinking is mitigated with a=0.20 , the two social births trigger a shrinking.

Figure 11 exhibits the subjective unit of time for the case of a person with two social births at ages 10 and 30. The power law has an exponent a=0.20 . Accordingly, as a function of time, the subjective unit of time is $U_{sa}$ for 0≤t≤10 , $U_{sa1}$ for 10≤t≤30 , and $U_{sa2}$ for 30≤t≤50 . Comparing with Figure 5 shows that the shrinking is mitigated with a=0.20 . But in contrast, having one more social birth increases the shrinking.

## 8. Conclusions

Starting from an observation about the perception of the passage of time, I have built a simple model using a sociophysics approach. Given an objective unit of time like the year, a mirror subjective unit of time is thus defined, from which a subjective duration is obtained. Three new functions arise naturally from it with the past horizon, the future horizon, and the speed of time with quantitative formulas. Soft and hard infinities are thus obtained with respect to looking to the past origin and the future end of a life.

I then introduce a first ritualized socialization, which adds a “second birth” and an associated second counting of the passage of time. The associated subjective unit of time is rescaled accordingly. Additional subjective clocks bonded to more ritualized socializations are stacked to shape a subjective unit of time, which exhibits a series of singularities.

The associated equations showed that the price for the first ritualized socialization is to exit a previous initial subjective feeling of eternity in terms of a future to be lived with the simultaneous reward of experiencing another moment of infinity similar to the birth.

Noticing that the amplitudes of the subjective units of time sound too small, I extend the inversely proportional dependence on the total number of passed objective units to a power law. The qualitative results are conserved with a simultaneous damping of the shrinking of time. The value of the associated exponent is a fitting parameter, which allows a possible connection to a quantitative exploration by allowing for some flexibility using the formula with a degree of freedom to be adjusted as needed.

Overall, the results recover common feelings about the passage of time over a lifetime, including:

- Before the first ritualized socialization, the future horizon is imperceptible, with the sensation of an endless life to be lived (soft infinity). After, the future horizon becomes finite and visible, yet once having experienced an infinite duration of time during the completion of the first unit of associated rescaled subjective unit of time.
- The past horizon is not localized with the sensation of a start from an infinite time ago (hard infinity).
- With the passage of objective time, the perception of the duration of time gets shorter and shorter. Concomitantly, the perception of the speed at which the time passes gets slower and slower.
- For young people, time flies and duration is long, while for old people, time gets almost frozen but duration is short. Intuitively, this paradoxical result can be comprehended with the case of a traveling car. Driving at a subjective speed during an objective duration yields the travelled subjective duration. The slower is the speed, and the shorter is the elapsed subjective duration to travel over an objective duration of time.

It is worth stressing the results have achieved the articulation of generality and independence of histories and locations of individuals. That goal is obtained using the birthdate of a given individual in a generic mathematical formula. The same holds true for past horizon, future horizon, and speed of time, which all use a generic formula in which the age of a given person is taken into account as well as the moment of those evaluations.

The model, despite being simple, yields a coherent frame for the dynamical measuring of the subjective perception of time. At present, it opens a new formal avenue to tackle the issue within a sociophysics approach but does not make direct contact with the considerable psychological literature on subjective time and the subjective passage of time [26,27,28,29]. For the future, identifying specific experimental settings to test and possibly adjust the model formulas could be a promising prospect to further understand this phenomenon.

## Data Availability

No data have been used in this study.
